# Young migrant men managing risk and seeking protection in a small town in Southern Uganda

**DOI:** 10.1016/j.jmh.2023.100191

**Published:** 2023-06-17

**Authors:** Edward Tumwesige, Allen Asiimwe, Rachel Kawuma, Sarah Bernays, Janet Seeley

**Affiliations:** a MRC/UVRI and LSHTM Uganda Research Unit, Entebbe, Uganda; b Department of Global Health and Development, London School of Hygiene & Tropical Medicine, London, UK; c School of Public Health, University of Sydney, Sydney, Australia

**Keywords:** Migrants, Youth, Sexual health, Poverty, Intersecting risks, Precarity

## Abstract

Young migrants in resource-constrained settings face multiple challenges when they move away from home for work and try to make their way in a new place. In Uganda, with a growing youthful population increasing numbers of young people are leaving home to look for opportunities in urban areas, often facing a precarious existence as they try to make money. Using data from in-depth interviews we investigate the lived experience of precarity of 20 young men who had recently migrated to a small town in south-west Uganda. We adopt a case study approach to look in-depth at the experience of three of the young men, showing how they engage in a continual evaluation of risk in their day to day lives, as they face multiple challenges related to their insecure employment and poor access to health services. We found that the risks that the young men are willing to take to maximise their limited opportunities changed over time. Our findings provide valuable insights into the gendered risks faced by young male migrants and illustrate the ways in which young migrants, many of whom may only have travelled a relatively short distance from home, face risks and challenges to their health and wellbeing, and need to be recognised as a population in need of attention and support.

## Introduction

Adolescent labour migration in Uganda is common; for many young people it has become a part of growing up. Young people move because they aspire for a better life for themselves, but they also move to make some money to help the family out, or at least contribute by reducing the number of mouths to feed at home (Rietveld et al., 2020). In Uganda that ‘new life’ may begin by being sought at a fishing site, a trading centre or in a bigger town or city, where a young person hopes to build a livelihood in fishing, repairing vehicles, selling foodstuffs, doing domestic work or serving in a bar or restaurant before, perhaps, moving on to a bigger place and greater opportunities (Barford et al., 2021). The move away from home may not be very far, the young person may go to a nearby town or trading centre, nor may that move be permanent (Rietveld et al., 2020). However, even if the rural home is a relatively short taxi or bus ride away, it is still a move away from parental supervision, offering a taste of independence. Yet, for all the optimism which may accompany the decision to leave home, particularly when that move is away from poverty, a move away can also mean an encounter with more acute precarity, if a job cannot be found or wages are not paid, or new friends exploit a newcomer’s naivete, leading perhaps to sexual exploitation or expensive gambling bills (Bwambaleet al., 2021; Mirembe et al., 2019).

We may think of illegality and deportability in the context of international migration, but internal migrants may need identity documents and reference letters/support letters in order to secure a place to stay and a chance at a job; in Uganda those letters are often required of young migrants, written by a local government official in their parental/rural home. Insecurity in employment and livelihood may be associated with illegality if those papers required to access work, or secure accommodation cannot be produced when demanded. Those without may join those in the poorest work conditions in the most insecure accommodation, unable to access any public services, if they exist.

Paret and Gleeson (2016) observe, for this reason, that the notion of ‘precarity is especially relevant to migrant populations’ (p.280). Millar(2017) explains that Bourdieu used the word ‘precarity’ to refer to precarious work ‘characterized by job insecurity, temp or part-time employment, a lack of social benefits, and low wages’ (p.3), and she goes on to note that youth unemployment (or indeed under-employment) prevents young people from getting on with their ‘life plans’; future-seeking becomes a dream rather than a plan towards a successful adult identity. Philo et al. (2019) conceptualise this precarity as pushing some to the socio-spatial margins, suggesting that the “making and possible unmaking of precariousness” can become “an injurious way of being-in-the-world” (p.150).

Kabiru et al. (2012) in their work with adolescents in informal settlements in Nairobi, explored the factors which supported young people to “make it,” that is, progress successfully through adolescence despite living in adverse conditions’ (p.13). They build on the ‘problem behaviour theory’ developed by Jessor (1991) to tease out the factors that result in some young people in their study setting in Nairobi being more resilient when faced with a precarious employment and living situation. Kabiru and colleagues developed a ‘protection-risk’ conceptual framework made up of three types of ‘risk factors’ (models’ risk, opportunity risk, vulnerability risk) and three types of ‘protective factors’ (models protection, controls protection, support protection). As the titles used by Kabiru et al. imply - a ‘model’ could be a good influence, promoting good behaviour or someone who might promote health compromising behaviour (practising unsafe sex, alcohol or drug misuse). Equally, opportunities may expose a young person to risky situations, working in a bar, for example, where it is expected that additional income can be made from providing sex or working in a workshop without protective equipment or clothing and without any oversight on safety. In such settings, protection can come from employers or older migrants who may provide advice and support, offering a safe place to shelter or stay. That support and resilience may also come from the young person themselves, perhaps through their religious faith, or a moral code instilled from childhood, although this may be threatened by the challenging conditions in their altered environment. Kabiru et al. conclude that those who ‘make it’ in the communities they worked in often had peers who served as role models providing examples of successful ways of earning a living, to stay safely and securely and to build supportive relationships.

However, for many young people moving from risk to protection is not a smooth progression, even when help is on hand. The notion of precarity (and more broadly precariousness) is important in understanding how fragile progress to ‘making it’ can be (Gukurume, 2018); in this paper we conceptualise a life-world that is in flux as we trace the fortunes of three young male migrants during their early months settling into a trading centre on a main highway, in south-western Uganda.

While a number of studies show that the chances of securing self-employment and casual work may be greater for young men than young women (Boutin, 2018; De Weerdt and Hirvonen, 2016; Nzabonaet al., 2019), and young women are particularly vulnerable to violence and sexual exploitation (Dambach et al., 2020; Logie et al., 2019), young male migrants also face particular challenges finding their way away from home (Kamara et al., 2019), facing risks to their security and health if they struggle to find paid work and make enough money to eat or find a safe place to stay. We identified that within our data several young men were exposed to sexual health risks which we had, prior to this study, associated more with young female migrants, drawn into sex work and exploitative relationships (Dzomba et al., 2019; King et al.,2021; Mojola and Wamoyi, 2019) and present a case study analysis of their experiences of precarity.

## Methods

In 2017-2018 we undertook a qualitative methods study with young people (aged 16-24 years old), who had recently moved to a trading centre in south-western Uganda, in search of work. We set out to understand these young people’s experiences as they managed the search for work, secured a place to stay, and looked after their health and wellbeing in a new place, away from home. We used a longitudinal design, consisting of repeat waves of data collection over a year, so that we could capture changes over time in their lives while also nurturing trust between us and the participants.

We focused on young people who had moved to the trading centre in the last nine months, when the study began. Initial rapid ethnographic formative research enabled us to get an overall picture of the numbers of young migrants in the place, the work they were doing (or hoped to do) and the places they stayed, as well as the services they might access (health centres, for example). We chose a small number of potential participants during this time, paying attention to a mix of gender and ages. Those young people helped us to make contact with new migrants like themselves at their places of work and recreation, which enabled us to recruit 40 young migrants (20 women and 20 men). The recruitment into the study, and all data collection, was carried out by two social science researchers, who were relatively close in age to the young people in the study.

We conducted initial in-depth interviews with 40 young people. The interviews were conducted at the participant’s place of choice, guided by a topic guide covering a brief history of their move away from home, their experiences since arrival of work, accommodation and food provision, friendships and any challenges. The interviews were audio-recorded with the participant’s consent and lasted up to one hour depending on how much information the participant chose to share. The interviews were conducted mainly in Luganda, the main language in the study area. A few interviews were conducted in other languages such as Rufumbira, Runyankore, and Kinyarwanda. The interviewers were proficient in all these languages. Brief notes written during the interview, noting things that may not be captured on the recording, or when a participant asked the recorder not to be used, were written up in detail immediately after each interview.

We followed up 20 of the same young people, in a second individual interview about six months later to understand their lived experiences over time. We had always intended to conduct follow-up interviews with a smaller sample of young people, anticipating some loss to follow-up as young people moved away. Indeed, the choice of the 20 people who took part in the second round of interviews was influenced not only by their interest in staying in the study but also by who was still resident in the area. Indeed, several young migrants had moved on in their quest for work and were no longer available. In the second interview, for the young people we could locate and who were willing to take part, we built on the information gathered in the first interview to find out what had happened in their lives.

The two interviewers were in the trading centre very frequently over the course of the study, which enabled them to have informal conversations with many of these young people which allowed us to keep in touch. Some young people reached out to the two interviewers for advice, particularly with questions about health care.

We (the authors of this paper, which include the two interviewers [first and second authors]) conducted an iterative thematic analysis, discussing the emerging data in weekly meetings. This process fed into ongoing recruitment, sampling and revising the choice of topics to talk about during interviews. The discussions also formed the basis of analytical memos in which emerging themes were identified and explored. We summarised audio-recorded data into detailed interview scripts in English using a mixture of reported speech and verbatim quotes (Rutakumwa et al., 2019). Scripts were coded initially using an open-coding approach, then using a coding framework. Coded data were checked against themes identified during the team’s regular analytical discussions. Emergent themes were checked against the analytical memos and discussed by the team to ensure accuracy of representation.

In this paper we focus mainly on the experience of three young men from amongst the 20 with whom we had stayed in contact for more than six months and were amongst the 10 out of the 20 who participated in two interviews. We use a case study approach in which individuals’ trajectories can be presented in detail, including how they change over time, selecting examples that were representative of other young male migrants, exemplifying the experience of other young people we met (Yin, 2009). The intention of this form of case study is to distil the circumstances and experience of ‘a broader category of which it is a member’ (Bryman, 2016p.56). By providing detailed accounts of each of the three young men’s experience we are able to highlight the risks young people like themselves face, as well as the ways in which risks and opportunities are calculated and recalculated as each manages the precarity of their situation as they set out to ‘make it’.

Ethical approval was granted by Uganda Virus Research Institute Research Ethics Committee (Ref: GC/127/17/04/593) and the Uganda National Council for Science and Technology (Ref: SS4298). Participants were approached and invited to take part by the local research team and asked to provide written consent to participate in the study activities. Following Uganda National Council for Science and Technology guidance (UNCST, 2014), participants aged 16-17 years old, living independently and providing for themselves financially, were considered emancipated minors and able to give their own consent without requiring approval from a parent or guardian. Pseudonyms are used in this paper to protect the identities of the participants.

## Findings

The 20 men were aged 17 to 23 years old when we conducted the first interview with them. For 11 of the young men, the trading centre was the first place they had migrated to since leaving home. The other nine had worked in other towns before coming to the trading centre; they had moved because of difficulties with their work or accommodation in their previous location and had often moved based on information they had received from others about the availability of work in the trading centre.

It was apparent from the accounts that the young men gave of their move that they were navigating their way in a shifting social environment over which, at times, they had little control as they sought to find ways to succeed. Much of the literature on boys/men and migration focuses on the quest to get paid work. We found, that in addition to the quest for paid employment, each of the young men had a range of other strategies to try and get money and make their way in the new place.

All the young men were making some money when we interviewed them, although most did not have a guaranteed wage. Four were trainees in metal works/motor mechanics shops, eight were selling food items at the road toll on the main road that ran through the town, while two others made and sold chapatis (flour, oil and water pancakes) on the streets in the town. Two were fishing at a nearby fishing site, one was working at a rice farm, one was working in a video shop, and one was a cleaner in a guest house. All had aspirations for the future ‘when they had money’, often expressing vague plans about moving on to a bigger town where they could set up a business, build a house and settle down.

The three young men who are the main focus on in this paper, who we call Bosco, Davis and Isaac (not their real names), provide, through the accounts of their experience, insights into the challenges all the young men faced moving to a new place. Most of the young men had moved from places within southern Uganda and about half, including Bosco and Davis had moved less than 50 km from their homes. Others, like Isaac who had travelled more than five hours on public transport from his family home, had come from more distant places. Two of the 20 young men came originally from Rwanda, although both had been working in another town in Uganda before they moved to the trading centre. The three young men we focus on in this paper told us about growing up and their reasons for moving; accounts that echoed the experience and migration journeys of all the young men. Below we focus first on the experience of growing up before describing the risks the young men faced, and the ways they understood how they might protect themselves.

### Growing up

Bosco, Davis and Isaac had all grown up in rural areas, and like all the young men, told of families where money was sometimes scarce, which had affected their education and prompted them to leave home. Bosco and Davis both attended secondary school up to form four (equivalent of reaching ordinary level, the first senior level school leaver qualification). Bosco had been fortunate and had been awarded a scholarship by his local church to attend school, while Davis was supported by his father. Isaac did not complete primary school, because his father had many children by different women, and the family struggled to find money to support him and his siblings to attend school.

Bosco and Davis both had a dream of joining the armed forces when they were in secondary school. Their model job was that of a soldier or a policeman. Davis talked of his love of guns and his continued envy of people with jobs that allowed them to ‘hold a gun’. The parents of both stopped their sons from trying to join the army and police; both said they were told by their families that those jobs were too risky. Instead, their families helped them find ‘apprenticeships’, unpaid trainee posts, in workshops in the trading centre, which is how we met them.

Isaac had a different experience from the other two as a child. His-parents had separated, and he found himself without support from his family. He told us that he often thought that if his parents had stayed together, he could have completed his education, reflecting that his life could have been very different. He learnt to fish from the age of 11 years in the nearby Lake Victoria to try and get money so he could stay in school. However, by the time he was 14 he decided he wanted more, so he dropped out of school and left his rural home to get a job as a crew member on a fishing boat based on one of the islands in the Lake. He recounted with pride how he made a lot of money in a short time and ‘his life changed’. He bought a piece of land, while he was still a teenager, but had no money to develop it. Through a friend he secured a more lucrative fishing job with the skills in fishing that he had acquired. The place he had moved to was on the mainland, close to a large town, where he used his money to enjoy ‘night clubbing’. He explained that he did make enough money to buy some more land. However, he still had nothing left to spend on developing that new land or his original piece of land. So, he had his land, but he had no other investment or savings, which he told us he regretted.

Working in fishing ended for Isaac in 2017, when the Government cracked down on illegal fishing, and Isaac lost his job because the boat owner was involved in illegal fishing practices and lost his equipment. Isaac, at the point, moved to the trading centre where he could still fish in the nearby Lake but could also supplement his earnings with other casual work.

Bosco and Davis benefitted from family support to get their first chance to learn a skill when they left school: metal fabrication. Both, it could be argued, had been protected by their families from the risks that their parents associated with jobs in the military and police, and directed to opportunities closer to their rural homes. Isaac had no such support and claimed he had been used to looking after himself from an early age because of his parents’ separation; indeed, until he lost his lucrative fishing job, he had been doing very well building his own career. Growing up near Lake Victoria he would have known of others who had made a good living from fishing and had followed their example when he moved to the island to fish.

### Opportunities and risks

Bosco’s father’s friend got him a vocational training opportunity at a metal works in the trading centre. Davis also got a place in another metal workshop through a friend of an uncle who also provided a place for him to stay for the first few days of his stay. When they first moved away from home their parents had helped both with money for food, but that support was limited and ended after a few weeks. Bosco lamented the poor accommodation he was given at the metal workshop, which he shared with other trainees, and the struggles of his early weeks. Davis, who could have stayed with his uncle’s friend, opted to get his own accommodation with fellow trainees because he wanted to be independent of his family. Both talked of ‘escaping’ from parental support and making their own way. Without parental support Isaac had already grown accustomed to making his own way before he had come to the trading centre through fishing.

Money for food and accommodation was a worry for all three young men, as it was for all the young men we interviewed. Bosco and Davis received no pay for their work at the workshops even though they had initially been promised a modest income from the sale of the items they produced as they trained. Indeed, several of the young men explained that those they worked for considered them ‘apprentices’ who needed to gain skills before they could be paid. Bosco explained that the trainees at the metal works survived by getting small tips from customers and on Sundays they would go to nearby farming areas and offer their labour in return for food or cash. Davis had a similar strategy to get money − he spent the first few hours of each day doing casual labouring to earn before going to his unpaid trainee job at the workshop. Isaac got some money from fishing, but he too helped on farms nearby during busy periods and sometimes got casual work at a large rice farm just outside the town. The three young men spoke with pride about the way they had managed to look after themselves, but it was clear that life was often a struggle, with Bosco and Davis recognising that the long hours they spent at their respective workshops working for no pay was exploitative. They, like all the young men, recognised that they had no alternative until they had enough skills to get a paid job and move on. Amidst the pride with which they talked about getting by, there was also mention of struggle. Bosco explained how he managed: “… I have boiled water in my room and on several occasions when I have failed to get money to buy anything small to eat, I have taken this water and slept”. Bosco’s lack of food was a common problem mentioned by other young migrants we spoke to; several talked of depending on one meal a day or even a single meal for two days. Constant hunger was a familiar problem.

The study’s longitudinal design enabled us to follow these young men’s experiences over time. During the second interview, six months after the first, Bosco was still at the same metal works, still training, and still not being paid for the work he did there. He complained that the challenges he faced had not eased, with the continued absence of food, accommodation, pay and inadequate training offered by his ‘position’. Even so, he still hoped for a better future.

Isaac seemed better off, perhaps because of his greater experience of getting by on his own. He said he got cheap food from one of his girlfriends who sold cooked food in the town. Such relationships, which several of the other young men talked about (sex in return for food) were something all three young men sought. Bosco and Davis took some time to establish sexual relationships in the trading centre; the delay, which they both lamented, may have − ironically − been protective in terms of sexual health.

### Protection from risk

None of the 20 young men could afford to go to sex workers when he first arrived in town, nor could any of them establish a steady relationship with women their own age. They complained that a girlfriend expected money from a partner, which they did not have, and they did not have money to pay a sex worker. However, a number of the young men told us that a sex worker was cheaper than having a girlfriend. Davis, for example, said he had tried to go out with a girl who he liked when he first arrived but: ‘[it] didn’t go far. I had no money to look after a girl; I could not afford money for lodging because I was sharing a room with a colleague’. Bosco managed to start a relationship with a school girl in the town who did not expect money from him, he said she understood his financial situation. Sadly, for him, her parents moved her to another school and the relationship ended, he said ‘I would have gotten another girlfriend […] but [given] the economic situation today I cannot … because every girl you get today wants money’.

Isaac explained that he did eventually get money so that he could go to a sex worker in the town. He said he found the sex worker ‘came with condoms’: she kept a supply for clients to use. He established a friendship with one particular woman, who then offered him discounted services. He said he always used a condom with this person, even ‘when we became friends.’ Isaac talked about other young men in the town including some of his friends who rarely used condoms ‘when they bought sex from commercial sex workers’ because they wanted ‘value’ through having ‘skin to skin’ sex. Isaac said ‘they ask you how they benefit from using a condom, yet they have paid money to enjoy sex’. Isaac also thought that the reluctance of ‘other men’ to use condoms was because they did not know how to put them on and so they had to rely on the sex worker to ‘open and dress them’.

Despite the sex workers having a supply of condoms, which some insisted on using with clients, many of the young men we spoke to believed that the risk of becoming infected with HIV was higher when sleeping with sex workers than their peers or younger girls. Yet some of those young women also had relationships with older men concurrently. Davis, for example, eventually established sexual relationships with two young women of his own age who were at the same time in relationships with older men who gave them money for cosmetics and to get their hair done in a salon; this was a good arrangement for Davis because, as a result, they did not ask him for money.

The new migrants recognised that the economic status of a young man was the main influencing factor on whether they had access to sex, either commercially or through relationships. All wanted to establish a long-term relationship with a girlfriend they could trust.

### Risks in tension

The 20 young men talked of risks from not being able to eat properly and poor working conditions. Bosco, for example, described not having access to protective equipment for welding in the workshop where he was learning a trade. However, all said that it was their sexual behaviour which posed the greatest risks, through infection with HIV and other sexually transmitted conditions. Each one of the young men spoke of the greater care they took when they compared themselves to ‘other’ young men; social desirability perhaps influenced by their perception of what was good behaviour in the eyes of the person talking to them. They rationalised their approach to getting access to sex as being mindful of risk and, as Isaac’s example above suggests, asserted that they took some preventive measures.

Bosco reported that while migrants like himself could appreciate their independent life away from the watchful eye of caregivers, it could concomitantly also ‘spoil’ life. He described how his friends have misused this ‘freer life’ with many being arrested for petty crimes for stealing or drug use. Bosco, Davis and Isaac and the other young men we interviewed, were quick to condemn ‘other young people’ who misused their freedom to engage in the overuse of alcohol and drugs and, in Bosco’s words ‘engage in reckless sexual activities with commercial sex workers, older women [which meant any woman older than them, probably in their late 20 s or 30 s] and widows for little return in the form of money and any other material gain’.

Davis talked about how many migrant young men would put their lives at risk by getting into relationships with older women because they were economically desperate. That said, when we first met Davis, he talked enthusiastically about getting into a relationship with an older woman. He said: ‘I know a friend who has a baby with an old woman whose husband passed away in an accident [.] she has enough money, so she looks after him and their baby’. Davis went on to say: ‘I will not lie, if I got an older woman who is very rich and willing to give me the money I want, I would go and respond to her sexual demands’. He reasoned: ‘When she is giving me enough money to do all I want? I would do it (have unprotected sex) because even being HIV negative but poor is not good at all. I would rather be positive but rich’.

While several young men told us about ‘others’ who had relationships with older women, it was only Isaac who was open when we first met about actually having a relationship with a woman who was older than himself. He explained that he had sex with an ‘older woman’ who had helped him a lot when he had first moved to the trading centre. He said ‘I have loved an older woman and ever since I met her, my financial status changed because in her are my blessings. I had bought a plot of land and could start building a house in my village’. That relationship had ended, but Isaac recalled fondly the benefit he had derived from having sex with that woman.

Davis had given up the idea of establishing a relationship with an older woman after six months living in the trading centre. By then he had found a way to make some money for himself:

‘I no longer admire that [having an older rich woman]. What brings about such kind of plans is poverty. When you are not poor you cannot think about those things […] I am lucky this did not happen to me and now there is no way I can love an older woman. Young boys love older women not because they want but simply because of poverty”.

Davis’ concern about these relationships was because of disease. He went on to say ‘They (young men) are likely to contract diseases like HIV, syphilis and others simply because the older they (women) are, the more they have slept with many people in their lives’. However, both he and Isaac had unprotected sex with their peers, young women who were in relationships with older men. Davis, as noted above, enjoyed his relationship with two young women because they did not ask him for money. He admitted to having unprotected sex with them even when he did not know their HIV status. He claimed he did not want ‘to bother’ about their status, because he would have to go and test for HIV. He reasoned: ‘If I tested and was found [HIV] positive I can die of thoughts but without testing I live normally and do my work without any thoughts’. He, and other young men, were concerned about the risks of sex with older women who they needed for support when they were struggling financially, but once they had some money of their own, they enjoyed unprotected sex with women of their own age and put any concerns about risk to one side.

### Choosing risk over protection

Protection, in the terms of the Kabiru et al. (2012) protection models, controls and support, was available to young men like Bosco and Davis. Bosco did have friends of his family nearby; the ones who had helped him when he first came, but he did not seek out help, nor did he go home when things were difficult. He was eager to make his own way. Davis’s story of choosing to live with his work colleagues, rather than with his uncle, reflects the situation of other young migrants we met who reported enjoying sense of freedom by choosing to forego free accommodation and food that might have been provided by relatives or friends of their family. Only four out of the 20 young men stayed with a relative or an employer. While all the men wanted independence, they admitted that they did not find life very easy. Davis described the life after leaving his father’s friend’s home as a ‘hassle’; but he managed for a while. He did go home, briefly, when he was struggling for money once he had done some months of his training. He returned though, when he had secured a job as a welder in town. He was proud that he was not having to stay at home and, importantly, depend on parents.

Other young men we spoke to talked of seeking accommodation with their friends, often of poorer quality than that offered at their place of work or with family friends, because of the lack of restrictions on what they did and the privacy they felt they gained away from the watchful eyes of those who might disapprove of their behaviour.

## Discussion

The experience of Bosco, Davis and Isaac illustrates the mix of factors which allowed them to ‘make it’, or at least be on the way to making it. All those we interviewed looked forward to a better future. Their stories show that the risks and protective factors described by Kabiru et al.(2012) are often mixed up: a sexual relationship with an older woman may be risky, but if she provides money that might allow someone like Isaac to buy land then that may be protective by making his future a little more secure. Furthermore, if she was involved in the sale of sex and had a supply of condoms which she used with her regular partner, sex with her may be less risky than with a younger girlfriend who also had an older male partner. That said, claiming to use condoms all the time, is simpler than admitting to irregular use, something we know is increasingly likely if a couple have regular sex together and establish an emotional bond (Mbonye et al., 2022;Rutakumwa et al., 2015; Schmidt-Sane, 2021). The young men described relationships with older women as simultaneously strategically valuable and undesirable. Yet, they alluded to a hierarchy of risks in which protecting against abject poverty was more pressing than HIV infection, suggesting that the negotiation of decisions, shaped by relative risk and relative pleasure (as well as opportunities), is complex for young men and may have been under-estimated (Grimsrud et al., 2020; Okafor et al., 2018;Pulerwitz et al., 2021).

While the young men were vulnerable to sexual risk taking, they were also vulnerable to exploitation in their workplace; often receiving no pay in return for being trained. Young men were serving as unpaid labourers for the workshop owners. For Davis this hardship had given way to paid (casual) employment as a welder, but not until time had been spent struggling to get by. Isaac too, for all his pride in his achievements of making his own way from a young age, had seen his livelihood collapse when the person he worked for in the fishing business was stopped from illegal fishing practices. The days of making quick money from artisanal fisheries are dwindling in the wake of Government crackdowns and fluctuating fish stocks (Mpomwenda et al., 2022;Seeley et al., 2009), and Isaac was caught up in the changes taking place in the industry. Thus the precarity of the lives of these three young men, and others we met like them, can perhaps be described by using Vigh’s concept of social navigation (2009), which moves us away from a linear progression from precarity to security, to provide a way to combine the sense of precariousness with the notion of finding a way through the insecurity, through twists and turns as ventures succeed and ventures fail.

In his work with urban youth in Guinea Bissau, where socio-political turbulence removed everyday certainties, Vigh (2009) found ‘persistent hardship’ as young people struggled to find ‘the next meal, find the next job and survive the present, and an unceasing attempt to figure out a way of gaining viable life chances, social worth and recognition’ (p.421). In doing so, he observed that, young people look for opportunities, and move on, navigating their way through, looking for a ‘spatial fix’, what Hannam et al. (2006) refer to as ‘moorings’, as a form of temporary security. We can see the twists and turns in the lives of the three men we have focused on in this paper. Their moorings were not necessarily fixed in space; a mooring could be the knowledge of a rural home that could be returned to if things got particularly difficult or could come from their growing confidence through acquiring a skill set, which allowed them to make money and at the same time establish desired relationships. Perhaps for each of the young men in our study an important protective factor was not only someone to turn to or someone to help them navigate their way through life in the town, as proposed by Kabiru et al. (2012), but their own sense of pride in becoming ‘a man’, upholding prized masculine ideals through their potential earning power and their sexual prowess (Mbonye et al., 2021); ideals which may compromise their readiness to access sexual health services and manage their sexual risks (Siu et al., 2012).

Bosco and Davis’ parents had stopped them from joining the armed forces because those jobs were perceived to be too risky. Instead, they embarked on a quest to ‘make it’ in what their families had perceived to be the more protective environment of a local town; an environment characterised by a precarity which the parents themselves little understood. Isaac had no such guidance but ended up in the same place, aiming to (re)make his living. Each continued to aspire to a future with a secure livelihood. While they may not have faced the physical danger associated with an army or police role, ‘making it’ through these alternative routes was still risky in terms of their health and wellbeing.

The study’s strength lies in its in-depth examination of three cases which provide an understanding of the trajectories of young migrant men like themselves. However, because the study focused on a small town in southern Uganda, it is important to recognize that the findings are not intended to be generalisable to other regions or countries. A limitation of our study is the focus on one small town.

## Conclusion

The accounts of these three young men illustrate how each faced risks to their health and wellbeing in a setting of exploitative work practices, hunger, and risky sex in their pursuit of a ‘freer and better’ life away from home. This indicates that services need to be ready to engage with the gendered lived experiences of young migrants, recognising the specific precarity faced by men. In a quest for independence in a new place, support (on their terms) would often be welcome, to help them to navigate with minimal harm the entangled risks of poverty, relationships and sexual health. The findings from the study have important implications for approaches aimed at supporting internal migrant populations, including young people, in Uganda and elsewhere in Africa. Our findings suggest that young migrants would benefit from having someone they can trust to turn to for advice and help to support their health and economic well-being. Our findings also illustrate the ways in which young migrants, many of whom may only have travelled a relatively short distance from home, face risks and challenges to their health and wellbeing, and need to be recognised as a population in need of attention and support,
